# Exploring the Complex Relationship Between Antidepressants, Depression and Neurocognitive Disorders

**DOI:** 10.3390/biomedicines12122747

**Published:** 2024-11-30

**Authors:** Monica Neațu, Iulia Ioniță, Ana Jugurt, Eugenia Irene Davidescu, Bogdan Ovidiu Popescu

**Affiliations:** 1 Department of Clinical Neurosciences, “Carol Davila” University of Medicine and Pharmacy, 050474 Bucharest, Romania; monica.neatu@rez.umfcd.ro (M.N.); iulia.ionita@rez.umfcd.ro (I.I.); ana.jugurt@rez.umfcd.ro (A.J.); bogdan.popescu@umfcd.ro (B.O.P.); 2 Department of Neurology, Colentina Clinical Hospital, 020125 Bucharest, Romania; 3 Department of Cell Biology, Neurosciences and Experimental Myology, “Victor Babeș” National Institute of Pathology, 050096 Bucharest, Romania

**Keywords:** dementia, cognitive decline, depression, antidepressants

## Abstract

The coexistence of dementia and depression in older populations presents a complex clinical challenge, with each condition often exacerbating the other. Cognitive decline can intensify mood disturbances, and untreated or recurring depression accelerates neurodegenerative processes. As depression is a recognized risk factor for dementia, it is crucial to address both conditions concurrently to prevent further deterioration. Antidepressants are frequently used to manage depression in dementia patients, with some studies suggesting they offer neuroprotective benefits. These benefits include promoting neurogenesis, enhancing synaptic plasticity, and reducing neuroinflammation, potentially slowing cognitive decline. Additionally, antidepressants have shown promise in addressing Alzheimer’s-related pathologies by reducing amyloid-beta accumulation and tau hyperphosphorylation. However, treatment-resistant depression remains a significant challenge, particularly in older adults with cognitive impairment. Many do not respond well to standard antidepressant therapies due to advanced neurodegenerative changes. Conflicting findings from studies add to the uncertainty, with some research suggesting that antidepressants may increase dementia risk, especially when used in patients with undiagnosed early-stage dementia. This article aims to explore the intricate relationship between depression and dementia, examining the benefits and risks of antidepressant use. We highlight the urgent need for personalized, comprehensive treatment strategies that balance mental health improvement with cognitive protection.

## 1. Introduction

Dementia and depression, two of the most widespread and debilitating health conditions, pose a growing challenge as the global population ages [1,2,3]. Dementia is a broad term used for a spectrum of cognitive disorders marked by significant impairments in mental functions that disrupt daily life and activities. It involves a decline across multiple cognitive domains, including executive function, concentration, attention, communication skills, knowledge acquisition, memory, sensory–motor integration, and social cognition. This cognitive decline is not confined to mental tasks but extends to essential everyday activities. As the condition progresses, individuals may lose awareness of their deficits, leading to a significant loss of autonomy and posing additional challenges to effective care and management [1]. From an epidemiological perspective, dementia is a growing global health issue. Currently, over 55 million individuals worldwide live with dementia, and nearly 10 million new cases are diagnosed annually. By 2050, the prevalence is projected to rise sharply to an estimated 131 million, as life expectancy rises [1,4].

Depression is a complex mood disorder marked by persistent feelings of sorrow and emptiness, often accompanied by substantial physical and cognitive changes that impair daily activities. According to the Diagnostic and Statistical Manual of Mental Disorders, Fifth Edition (DSM-5), depressive disorders are classified into several subtypes, including disruptive mood dysregulation disorder, major depressive disorder, persistent depressive disorder, premenstrual dysphoric disorder, and depressive disorder due to another medical condition [5,6]. Major depressive disorder (MDD), the most common form, is diagnosed when a person displays a persistently low mood or disinterest in activities, along with five or more symptoms such as weight fluctuations, sleep disturbances, fatigue, trouble focusing, psychomotor changes, inappropriate guilt, or recurrent thoughts of death, lasting for a minimum of two weeks. For a certain diagnosis, these symptoms must result in notable impairments in critical areas of functioning [5,6,7]. Epidemiologically, MDD is a growing global health issue, affecting roughly 7% of the population over the period of a year. Its lifetime prevalence is estimated to be around 12%. The condition disproportionately affects younger adults aged 18–29, who exhibit a prevalence three times higher than that observed in individuals aged 60 and older. Despite the high prevalence of depression, the stigma surrounding mental health remains a significant obstacle that leads approximately 60% of individuals suffering from depression to avoid seeking professional help [1,2,3,4,5,6,7,8,9,10,11,12,13].

Antidepressants are often prescribed to treat depression, yet their safety and effectiveness in older adults with coexisting MDD and dementia continue to be a topic of discussion. Randomized controlled trials, which are considered ideal in clinical research, have shown mixed results. Some indicate that different antidepressants may provide limited relief from depressive symptoms without significantly worsening cognitive function, while others fail to demonstrate clear therapeutic benefits [12]. Moreover, observational studies, though useful in assessing real-world outcomes, introduce additional layers of complexity. These studies often reflect confounding variables such as the severity of dementia, preexisting comorbidities, and variations in individual responses to medications [12,14]. Such factors can independently influence cognitive decline, making it challenging to isolate the effects of antidepressants in this population [12,14,15,16,17,18,19].

The pharmacodynamics of antidepressants add another layer of complexity, which can interact with various physiological systems implicated in both conditions. For instance, certain antidepressants may offer neuroprotective benefits by modulating neuroinflammatory pathways and promoting neurotrophic factor regulation. However, others may exacerbate dementia risk through mechanisms unrelated to their efficacy in treating depression [12]. This duality raises critical questions about the right choice of antidepressant therapy in older adults, necessitating an individualized approach where both mental health benefits and potential cognitive risks are carefully weighed [12,20].

We consider that as the population of elderly individuals struggling with both depression and dementia continues to rise, addressing the ambiguities surrounding the use of antidepressants becomes increasingly urgent [1,2,3,4]. A critical reexamination of the existing literature and ongoing clinical practices is essential to guide therapeutic decisions that not only optimize mental health outcomes but also protect cognitive function [21,22]. We consider that it is necessary to have a careful and objective analysis of the implications of using antidepressants in people with dementia [23]. Therefore, we seek to explore the complex relationship between dementia and depression, illustrating how each disease can intensify the other, leading to an increased overall burden for patients. Furthermore, we aim to provide a balanced examination of both the advantages and the disadvantages associated with antidepressant treatment in the context of neurocognitive disorders [21,22,23,24,25,26,27,28,29].

## 2. Dementia-Induced Depression

It is well known that a progressive cognitive decline is the primary hallmark of neurocognitive syndromes. However, a great number of individuals with dementia also experience neuropsychiatric symptoms (NPSs) throughout the course of the disease, and these symptoms are now recognized as a fundamental aspect of the disease [30,31,32].

Mild cognitive impairment (MCI) involves an observable cognitive decline that does not yet interfere significantly with everyday tasks. MCI can be clinically categorized into amnestic MCI (aMCI) and non-amnestic MCI (naMCI), based on the primarily affected cognitive domains. In aMCI, characterized by notable memory impairments, there is a strong association with medial temporal lobe and hippocampal dysfunction. This subtype often presents with NPSs such as depression, apathy, and anxiety [33]. Depression might arise from an acute awareness of memory deterioration, while apathy is linked to disruptions in the motivational and reward-processing networks. Anxiety, commonly observed in aMCI, may result from hyperactivity in the amygdala and the dysregulation of the hypothalamic–pituitary–adrenal (HPA) axis, which heightens stress sensitivity [33,34,35].

On the other hand, naMCI affects cognitive areas other than memory, including executive functions, language, or visuospatial skills, depending on the involved brain regions, such as the frontal, parietal, or occipital lobes. In executive naMCI, symptoms such as irritability, disinhibition, and agitation are prominent, reflecting deficits in the prefrontal cortex that impair self-regulation and impulse control. In visuospatial naMCI, associated with parietal and occipital lobe dysfunction, symptoms like apathy or emotional blunting may emerge from a decreased interaction with a visually complex environment. Although less frequent than in aMCI, anxiety and depression can also present in naMCI [35,36,37,38].

The progression of MCI and its transition to dementia is marked by distinct stages, each characterized by specific NPSs [38]. In the earliest stages of MCI, depressive symptoms often dominate, probably driven by an individual’s awareness of cognitive decline, which may lead to sadness, frustration, or anxiety, especially of a generalized or anticipatory nature. As the condition progresses, irritability, apathy, and agitation emerge as neuropathology extends to the prefrontal regions responsible for self-regulation and motivation. These symptoms, along with sleep disturbances, become more prominent in moderate stages of cognitive impairment [38]. Nearing the threshold of dementia, more severe NPSs such as paranoia, delusions, and hallucinations surface, stemming from widespread cortical and subcortical disintegration and resulting in the increased misinterpretation of external stimuli. In advanced stages of dementia, psychotic features such as persistent hallucinations, delusions, aberrant motor behaviors, and even sexual disinhibition become prevalent. While early-stage symptoms like depression and apathy are often long-lasting, later-stage symptoms such as hallucinations and aggression exhibit shorter persistence but greater intensity, highlighting the evolving and dynamic nature of NPSs across the spectrum of cognitive decline [33,34,35,36,37,38].

When it comes to depression, it is observed in up to 85% of individuals diagnosed with MCI. Researchers have extensively examined whether there is any relationship between depression and the evolution from MCI to dementia. On one hand, we found that some studies reveal that the severity of NPSs in individuals diagnosed with dementia has been linked to a more rapid cognitive deterioration. On the other hand, recent prospective research has revealed no significant connection between the two. Specifically, four recent studies found no substantial evidence supporting the idea that depression accelerates the transition rate from MCI to dementia. As a result, the current body of evidence remains inconclusive regarding the role of depression in this context [38,39,40].

The pathophysiological mechanisms linking NPSs and MCI are multifaceted and interconnected. Neurodegeneration in key regions such as the hippocampus, anterior cingulate cortex, and amygdala disrupts cognitive–emotional circuits, impairing not only memory and executive functions but also emotional regulation. At the molecular level, shared pathological hallmarks of neurodegenerative diseases, such as amyloid-beta plaques, tau tangles, and neuroinflammation, contribute to both cognitive deficits and NPS [38]. Moreover, neurotransmitter dysregulation serves as a critical link between MCI and NPS. Cholinergic deficits impair attention and memory while also contributing to apathy and reduced emotional engagement. Monoaminergic systems, including dopaminergic and serotonergic pathways, are similarly affected, with disruptions in these systems correlating with depressive and anxious symptoms. Importantly, the interplay between NPSs and MCI is bidirectional; the presence of a NPS itself may accelerate MCI progression. Behavioral symptoms such as apathy or irritability may exacerbate social withdrawal, physical inactivity, and poor sleep, all of which are independent risk factors for cognitive decline. Furthermore, we will explore the pathophysiological mechanisms involved in the development of major depressive disorder within the context of neurocognitive disorders [35,36,37,38,39,40].

### 2.1. Potential Mechanisms for the Development of Depression in Individuals with Existing Neurocognitive Disorders

#### 2.1.1. Protein Aggregation

It is well established that protein aggregation is a hallmark of most dementias, playing a causal role in the progression of the disease. While these proteins have been widely studied regarding cognitive decline and dementia onset, their relationship with NPSs like depression remains less explored. We consider that investigating their potential connection to depression in conditions like MCI or dementia could be insightful [30].

In Alzheimer’s disease (AD), the interaction between amyloid-beta (Aβ) and depression remains unclear, primarily due to inconsistent findings across studies [32]. While some research has indicated that the existence of amyloid plaques is correlated with MDD, it does not correlate with increased depressive symptoms. Additionally, investigations of individuals who have developed dementia found no significant links between depression and markers such as amyloid plaque accumulation, neurofibrillary tangles, or hippocampal sclerosis [32,41,42]. Consequently, many researchers argue that late-life depression (LLD) is not directly connected to dementia-related pathology in AD. However, studies examining cognitively normal individuals with LLD have identified associations between pathological forms of Aβ and depressive symptoms, suggesting that early AD pathology could potentially trigger depression in some cases. In instances where MCI or AD is already diagnosed, isolating depression as solely related to AD pathology may prove insufficient, given the extensive burden of disease that manifests as broader cognitive and behavioral symptoms [32,41,42,43,44,45,46,47,48].

We find that the connection between Lewy Body pathology and depression in dementia is notably more robust in the existing literature. Research consistently indicates that patients diagnosed with Lewy Body Dementia exhibit higher rates of depression than those with Alzheimer’s Disease. Investigations have revealed that significant Lewy Body pathology is often present in subcortical regions, areas associated with mood regulation. Furthermore, a particularly interesting study identified higher levels of alpha-synuclein in the plasma of individuals also suffering from MDD, suggesting a potential link between this protein and mood disturbances [32,49,50].

Research exploring the link between TAR DNA-binding protein 43 (TDP-43) and depression, particularly in the context of Limbic Predominant Age-Related TDP-43 Encephalopathy (LATE), remains sparse, with only a handful of studies investigating this relationship. One notable study revealed that these patients, over the age of 50 who were experiencing a major depressive episode, exhibited significantly more elevated levels of TDP-43 in their serum versus their non-depressed counterparts [32,51,52].

#### 2.1.2. Immune Mechanisms

The emergence of depression in subjects with preexisting dementia may be intricately linked to inflammatory processes. An increasing amount of evidence suggests that inflammation serves as a pivotal pathway in the relationship between dementia and depression, highlighting the need to understand how peripheral cytokines interact with biological mechanisms to contribute to both cognitive decline and mood disorders. Numerous studies have documented altered levels of inflammatory markers, both pro- and anti-inflammatory cytokines, and chemokines in individuals with dementia, MDD, and LLD [53,54,55].

Microglia are immune cells crucial for maintaining homeostasis in the central nervous system (CNS). In the context of dementia, microglia have been identified as key players that contribute to cognitive decline. They initially act to protect neuronal health by clearing harmful substances through phagocytosis. However, in a state of chronic inflammation often observed in dementia, microglia can become overactive, leading to detrimental effects on the normal function of synapses and the release of pro-inflammatory cytokines [32]. Research has identified a variety of markers in blood and cerebrospinal fluid (CSF), such as IL-1B, IL-6, TNF, IL-10, and IL-12, which have been linked to both dementia and mood disorders. For instance, one study found that higher serum levels of tumor necrosis factor-alpha (TNF-α) were correlated with an increased depressive state over a six-month observation period in individuals with AD [53,54,55]. This suggests that inflammation may exacerbate the mood disturbances seen in dementia patients, creating a feedback loop that can accelerate cognitive decline [56]. We also want to point out that this chronic inflammation may also directly influence mood by disrupting serotonin signaling, a neurotransmitter crucial for emotional and cognitive regulation. It has been described that pro-inflammatory cytokines interfere with serotonin production and uptake, contributing to depressive symptoms [32,53,54,55,56,57].

Moreover, external stressors, such as social isolation and challenges in forming attachments, can exacerbate the inflammatory responses mediated by microglia. Individuals with dementia often experience these stressors, particularly in institutionalized settings where separation from loved ones is common. Such experiences can trigger inflammatory processes that not only worsen cognitive impairment but also promote depressive symptoms [58]. Research indicates that chronic stress is accompanied by an increase in immature, pro-inflammatory monocytes that migrate to the CNS and exacerbate microglial inflammation. This inflammatory cascade can impair neuronal function and disrupt neurotrophic factors, which are essential for neuron growth and survival. Consequently, these changes can result in reduced synaptic signaling and impaired neurogenesis, both of which are associated with depression [32,58].

#### 2.1.3. Vascular Hypothesis

Vascular dementia (VD) is an important and increasingly recognized condition defined by cognitive deficits directly associated with significant cerebrovascular disease. This syndrome highlights the intricate relationship between blood-flow abnormalities in the brain and cognitive decline [59,60]. While VD can manifest as a distinct entity characterized by its unique pathophysiological mechanisms, it is also commonly observed in conjunction with other forms of dementia. This overlap leads to the frequent diagnosis of “mixed dementia”, which comprises multiple underlying causes, often including vascular components alongside neurodegenerative processes [59,60,61].

There is a pronounced incidence of depression following cerebrovascular events such as cerebral infarcts and subcortical lacunar infarcts. This “Vascular Depression” hypothesis asserts that disruptions in the corticolimbic and corticostriatal circuits are significantly impacted by the loss of white-matter tracts due to vascular events, leading to an increased vulnerability for developing depression [32,61]. This theory is also supported by the presence of white-matter hyperintensities (WMH) discovered on T2-weighted MRI scans. These WMHs are typically localized in the periventricular and subcortical white-matter regions and can occasionally extend into subcortical gray matter [62,63]. They are often associated with partial demyelination and axonal loss, along with increased levels of gliosis, further exacerbating cognitive deficits and depressive symptoms in affected individuals [32,61,62,63].

It is worth mentioning that studies have indicated that depression occurs more frequently in patients with VD than in those diagnosed with AD, suggesting a unique pathophysiological relationship between vascular health and mood disorders [32,64].

#### 2.1.4. Genetic Factors

A range of studies has investigated the relationship between genetic factors and depression in AD. Research, predominantly targeting the apolipoprotein E (APOE) gene, has indicated a potential connection among depression and the APOE e4 genotype. The Val66Met allele of brain-derived neurotrophic factor has also been identified as increasing the risk for depression in AD. Moreover, an association between the 2R allele of the dopamine receptor 4 (DRD4) and heightened scores for depression was discovered [30,65].

#### 2.1.5. Alterations in Neurotransmitters

Research suggests that monoaminergic alterations are implicated in both depression and different subtypes of dementia, but our findings have been inconsistent. Monoaminergic deficits, particularly involving the degeneration of the locus coeruleus and substantia nigra neurons, have been more constantly observed in Lewy Body disease accompanied by depression compared to AD [66]. Also, more recent findings suggest that a reduction in 5-HT1A receptors is associated with depression in AD, contrasting with Lewy Body dementia, where a higher density of 5-HT1A receptors is correlated with depressive symptoms. These results highlight the differing underlying mechanisms of depression in AD versus Lewy Body dementias. Additionally, the neurotransmitter changes seen in dementia are believed to contribute to the resistance to depression treatment; however, we will explore this topic in more detail in a later paragraph [30,66].

Lastly, we aim to examine another crucial aspect of the relationship between cognitive impairment and the risk of depression: whether depressive symptoms might arise as a psychological outcome of the awareness of cognitive decline. Patients who begin to notice early signs of dementia may become increasingly aware of their deteriorating cognitive function, leading to feelings of helplessness and despair. This awareness can provoke depressive symptoms as individuals start to manage the implications of their cognitive losses, such as diminished independence. The gravity of receiving a diagnosis that suggests an incurable and progressive illness can understandably lead to profound emotional distress, amplifying feelings of anxiety and depression [31]. While it is confirmed that elevated rates of depression are frequent during the initial and mid-stages of various forms of neurocognitive disorders, it remains challenging to determine whether these mood disorders are primarily reactions to perceived cognitive losses or if they are influenced by biological mechanisms intrinsic to the dementia process itself [31,67,68,69].

## 3. Depression-Induced Dementia

An intriguing review focuses on defining neurocognitive profiles in MDD. There are three hypotheses important for understanding the cognitive decline in depression: state, trait, and scar hypotheses. They are crucial for understanding the etiological development and clinical implications of cognitive deficits in MMD. The state hypothesis implies that cognitive deficits are influenced by and fluctuate with depressive symptoms. From this viewpoint, cognitive impairment tends to improve as affective symptoms diminish. The trait hypothesis implies that a preexistent state of neurocognitive vulnerability is present that contributes to an increasing likelihood of developing depression. According to this theory, the cognitive profile of affected individuals is stable over time and independent of the current clinical state, persisting even in the remission periods. Finally, the scar hypothesis affirms that, depending on the duration and number of depressive episodes, neurobiological changes cause irreversible cognitive impairment over time [70,71].

Late-life depression, occurring in older adults typically aged 60 to 65, has been the focus of numerous studies due to its complex relationship with cognitive health. Importantly, LLD is now considered both a potential risk factor for dementia and a prodromal symptom of neurodegenerative processes. Despite many inconsistencies, a comprehensive review of the literature suggests that LLD is statistically associated with a significantly higher risk of developing dementia. A two-to-five-fold increase in dementia risk has been noted for individuals with LLD, although findings vary due to methodological differences such as sample size and different definitions of depression [32]. LLD frequently presents clinical manifestations such as executive dysfunction, psychomotor retardation, anhedonia, and reduced motivation, all of which have been linked to neurocognitive decline. Moreover, the severity and frequency of depressive symptoms appear to play crucial roles in influencing dementia [58]. We identified a clear dose-dependent relationship, indicating that an increase in the number of depressive symptoms correlates with a higher risk of developing dementia. This correlation is particularly described in research linking depressive symptoms to an increased likelihood of AD, with the risk escalating with each additional depressive symptom [32,58,72].

Although the connection between depression and dementia has traditionally been examined through the lens of LLD, recent research is increasingly exploring the role of earlier-life depression as a potential precursor to dementia. Studies focusing on depression in young adulthood and middle-aged populations remain limited, despite these periods being critical for understanding the long-term risk of cognitive decline [31]. Recent longitudinal studies provide compelling evidence that depressive episodes occurring before the age of 60 substantially elevate the risk of developing dementia in later years. These studies have demonstrated that early-onset depression, particularly when it is characterized by frequent and prolonged episodes, is associated with a two-to-four-fold increased likelihood of developing dementia [73]. It has been suggested that the timing and persistence of depression may shape the trajectory of cognitive health and influence the specific subtype of dementia that eventually develops. For instance, the risk of developing Alzheimer’s disease appears to be more pronounced when depressive symptoms manifest in late life, while the likelihood of developing vascular dementia is significantly higher when depressive episodes occur both in mid-life and later life (Table 1) [31,73].

### 3.1. Potential Mechanisms for the Development of Dementia in Individuals with Existing Depression

#### 3.1.1. Vascular Hypothesis

Depression is closely connected to vascular mechanisms, with a great body of research indicating that patients suffering from depression encounter significant reductions in cerebral blood flow (CBF). This decline in blood flow is believed to stem from dysfunctions in cerebral autoregulation, which become more pronounced with age, increasing the brain’s susceptibility to hypoxia and ischemia. Emotional states can exacerbate these vascular issues by raising blood pressure and vascular resistance while lowering the heart’s pumping efficiency, further reducing CBF [58,83,84,85,86,87].

Beyond CBF reductions, depression is a significant determinant for cerebral small vessel disease, which is linked to the emergence of ischemic changes. Individuals with depression often display abnormalities in brain regions particularly vulnerable to small vessel disease, as seen in MRI findings that frequently show white-matter hyperintensities. These white-matter changes, often located in the frontal and subcortical regions, are predictive of both depression and cognitive decline [58,88].

It is worth mentioning that the heightened risk of developing vascular disturbances in a depressed patient can also be attributed to behavioral factors commonly associated with depression, such as smoking, poor diet, and physical inactivity [89]. Individuals suffering from depression may neglect their physical health, leading to the exacerbation of cardiovascular risk factors like hypertension and obesity. Moreover, the physiological changes linked to depression, including increased inflammation and alterations in the autonomic nervous system, can further contribute to vascular deterioration [90,91,92]. This interplay between depression and vascular health creates a cycle where the presence of depression not only influences vascular conditions but may also worsen overall cognitive health outcomes [89,90,91,92,93,94,95,96,97,98,99].

The pathways between depression and vascular disease are complex and bidirectional, as each condition raises the risk of developing the other [24]. This interplay suggests that while vascular disease may lead to depression, depression can also promote vascular complications, creating a self-reinforcing cycle between mood disorders, cerebrovascular pathology, and cognitive impairment [24,58,100,101,102,103,104].

#### 3.1.2. Immune Mechanisms

One of the main processes connecting depression to cognitive decline is the body’s immune response to prolonged depressive episodes, which trigger chronic inflammation. Individuals suffering from depression often show elevated levels of pro-inflammatory markers, including interleukins (IL-1β, IL-6), TNFα, and C-reactive protein (CRP) [58]. This inflammatory response not only worsens depression but is also crucial in the deterioration of mental functions. Persistent inflammation in the CNS can suppress anti-inflammatory mechanisms, allowing pro-inflammatory cytokines to proliferate within the brain. These cytokines interfere with important neurological processes, such as serotonin metabolism, neurogenesis in the hippocampus, and synaptic plasticity, which are vital for cognitive health [90]. The resulting disruption to these processes can help explain why chronic depression significantly raises the risk of cognitive deterioration [58,90,91].

#### 3.1.3. Hippocampal Responses to Cortisol

Research indicates that mice subjected to prolonged stress or synthetic glucocorticoid exposure exhibit similar characteristics to individuals suffering from MDD. In rodent models, extended stress or glucocorticoid treatment causes damage to hippocampal neurons, leading to memory impairments. This damage progressively worsens with ongoing stress or hormonal imbalances. These findings are particularly significant, as they mirror the dysfunction of the hypothalamic–pituitary–adrenal (HPA) axis observed in humans [72,73].

In individuals experiencing depressive symptoms, the HPA axis becomes hyperactivated, leading to an excessive release of glucocorticoids, particularly cortisol. This hormonal imbalance can profoundly impair the hippocampus, a region abundant in corticosteroid receptors, therefore rendering it more susceptible to the detrimental effects of glucocorticoid exposure. Chronic elevations in cortisol not only diminish the expression of glucocorticoid receptors within this area but also inflict direct damage on hippocampal neurons [72,92]. Cortisol-induced hippocampal damage manifests as a reduction in overall volume, decreased dendritic complexity, and diminished neurogenesis. This neuronal damage tends to accumulate over time, due to excessive glucocorticoid exposure. Consequently, the body’s ability to regulate the HPA axis is compromised, creating a self-perpetuating feedback loop, a process often referred to as the “glucocorticoid cascade” [73,93]. Some studies have noted that hippocampal volume loss correlates with a higher risk of cognitive decline among depressed subjects. However, the evidence remains inconsistent, with certain studies failing to establish a direct relationship between prior depression and reduced hippocampal volume. This discrepancy suggests that hippocampal atrophy alone may not fully account for the heightened dementia risk in individuals with a history of depression [72,73,92,93,94].

While the HPA axis and elevated cortisol levels provide valuable insights into the connection between depression and dementia, it is important to recognize that cortisol’s role extends beyond its effects on the CNS. Elevated cortisol has been implicated in multiple pathways that contribute to neurodegeneration and cognitive decline. One such pathway is the vascular hypothesis, which suggests that chronic stress and high cortisol levels can exacerbate vascular dysfunction, leading to impaired blood flow in the brain, as previously discussed [58,96,97]. Additionally, cortisol can influence the immune system by promoting a state of low-grade inflammation and can upregulate inflammatory cytokines, which in turn may contribute to neuronal damage and the progression of neurodegenerative diseases. Furthermore, cortisol is involved in the regulation of neurotrophic factors, such as brain-derived neurotrophic factor (BDNF). Chronic elevated cortisol levels have been shown to reduce the expression of BDNF, impairing neuroplasticity and further contributing to cognitive decline. In the following paragraphs, we will explore in more detail how inflammation and neurotrophic factors contribute to dementia, building upon the points briefly mentioned earlier [72,73].

#### 3.1.4. Amyloid Build-Up

Amyloid plaques, along with hippocampal atrophy, serve as defining features of Alzheimer’s disease. As we mentioned before, the accumulation of both β-amyloid and tau proteins leads to the development of neuritic plaques and neurofibrillary tangles, which are critical to the disease’s pathology. Notably, studies have shown that these pathological markers are present in greater concentrations within the hippocampus of AD patients who also experience depression [72,73]. This finding points to a potential link between depression and the progression of AD through the mechanisms of amyloid plaque formation, although the precise nature of this relationship remains not entirely understood [72,96]. One proposed mechanism connecting depression to AD is the heightened production of β-amyloid as a response to stress, which often intensifies during episodes of depression. Research utilizing animal models of AD indicates that elevated glucocorticoid levels can stimulate the formation of β-amyloid by increasing both the levels of amyloid precursor protein (APP) and the enzyme responsible for its cleavage [73,97]. Additionally, depression may exacerbate the accumulation of β-amyloid through its impact on the serotonergic system, especially during the initial phases of AD. Moreover, a specific entity of depression, characterized by a high plasma Aβ40/Aβ42 ratio, was described. It has been associated with impairments in memory and visuospatial abilities. This entity has led to the hypothesis that amyloid-associated depression could stand for either a preclinical or early-stage form of AD, or a risk pathway leading to AD development [24,72,73,95,96,97,98,99,100,101,102,103,104,105,106,107,108].

#### 3.1.5. Neurotrophic Factors

Another potential connection between depression and neurocognitive disorders lies in the concentration and activity of neurotrophic factors, particularly brain-derived neurotrophic factor (BDNF). It is primarily located in the CNS, with a more significant presence in the hippocampus. BDNF is widely recognized for its crucial involvement in neuronal plasticity, proliferation, and differentiation, indicating that it is essential for preserving CNS integrity and supporting overall cognitive function. Although the literature presents some inconsistencies, it has been described that BDNF signaling is impaired in rodent models of depression induced by stress, as well as in individuals diagnosed with depression. Moreover, insomnia is a common characteristic among individuals with depression, and research also indicates a link between BDNF levels and sleep disturbances. Specifically, patients experiencing insomnia have been found to have lower BDNF levels. In addition to BDNF, other critical neurotrophic factors disturbances have been described: transforming growth factor-β1 (TGF-β1), insulin-like growth factor-1 (IGF-1), and vascular endothelial growth factor (VEGF). Each of these factors contributes to neuronal health and may be involved in the mechanisms linking depression and neurocognitive disorders [72,73].

## 4. Pseudodementia

To further properly evaluate the impact of antidepressant use in MDD, it is important to distinguish between MCI as an early symptom of a neurodegenerative condition and as an effect of a depressive disorder that mimics dementia but is potentially reversible and not rooted in neurological degeneration [24]. Pseudodementia (depressive cognitive disorders) refers to cognitive and functional impairments that mimic neurodegenerative disorders but are secondary to neuropsychiatric conditions, particularly depression. Distinguishing pseudodementia from true cognitive disorders can be challenging due to overlapping symptoms like impaired concentration, reduced motivation, and attention deficiency [100]. However, factors such as abrupt onset, fluctuating severity, a background of depressive or manic episodes, and family history can help lean toward a pseudodementia diagnosis. Making the right differential diagnosis is key to pinpointing those who would gain the most from antidepressant treatment, especially considering that this condition could be reversible with proper care [24,100,101]. A systematic review of longitudinal studies of pseudodementia including 284 patients (of which 238 were diagnosed with dementia) showed that 53% of patients no longer fulfilled the criteria for dementia after treatment of their psychiatric condition, whereas 33% developed irreversible dementia at follow-up. In one research, 44 elderly individuals with pseudodementia were monitored for up to 18 years, with assessments every six months following the initial diagnosis of depressive dysfunction and the start of antidepressant treatment. Initially, cognitive function returned to pre-illness levels after treatment. However, by the end of the study, 89% of these patients had developed dementia [24,101,102,103]. Therefore, when evaluating the prognosis of pseudodementia, it is crucial to recognize that cognitive impairment can be reversible in cases of moderate-to-severe depression with antidepressant therapy. Nonetheless, in the long term, particularly for older adults, pseudodementia is often seen as a strong predictor of irreversible dementia, as noted by several authors [24,100,101,102,103].

## 5. Cognitive Advantages of Antidepressant Use in Dementia

Antidepressants, particularly those from classes like SSRIs and SNRIs, have shown great potential in mitigating cognitive decline in individuals with both major depressive disorder and early dementia [12]. A systematic review of 43 randomized controlled trials revealed that antidepressants offer cognitive benefits, likely due to a combination of neuroprotective mechanisms. Medications such as fluoxetine, moclobemide, and vortioxetine not only alleviate depressive symptoms but also promote neurogenesis, reversing some neurodegenerative processes. The positive impact of these drugs on cognition stems from their ability to modulate inflammatory pathways, increasing the production of anti-inflammatory cytokines while decreasing pro-inflammatory markers. For instance, fluoxetine and venlafaxine have demonstrated an ability to decrease inflammation, while fluoxetine and vortioxetine have shown efficacy in reducing oxidative stress, another contributor to neurodegeneration [90,104]. Notably, some antidepressants, fluoxetine, citalopram, and amitriptyline, have been implicated in reducing the build-up of β-amyloid plaques, which are closely linked to Alzheimer’s disease pathology [105]. This multifaceted approach supports the recognition of antidepressants as beneficial in managing cognitive symptoms of dementia and potentially slowing its progression, as detailed below (Table 2) [12,90,104,105,106,107].

### 5.1. The Role of Antidepressant in Neurogenesis

Depression, especially when untreated, is known to cause significant neurodegenerative effects, including cell atrophy and neuronal loss, primarily in the hippocampus. These detrimental changes have been well documented in animal models, where chronic stress and depressive-like states lead to hippocampal shrinkage and reduced neural plasticity. Luckly, these neurodegenerative effects are not irreversible. The administration of antidepressants, particularly selective serotonin reuptake inhibitors (SSRIs) like fluoxetine and monoamine oxidase inhibitors (MAOIs) like moclobemide, has been shown to stimulate neurogenesis by facilitating the proliferation of neural progenitor cells within the hippocampus [20,90].

This regenerative process is not limited to the generation of new neurons; antidepressants also induce significant neuroplastic changes, reshaping synaptic structures and enhancing the expression of synaptic proteins. These neuroplastic effects are deeply intertwined with the brain-derived neurotrophic factor (BDNF) signaling pathway, specifically the BDNF-TrkB (tropomyosin receptor kinase B) cascade. This pathway plays a central role in supporting neuronal survival, dendritic growth, and synaptic connectivity. Antidepressants enhance BDNF activity, which subsequently activates intracellular signaling pathways such as the mitogen-activated protein kinase (MAPK) and phosphatidylinositol 3-kinase (PI3K/Akt) pathways. By restoring MAPK activity, antidepressants improve dendritic complexity, thereby enhancing the brain’s capacity for learning, memory consolidation, and emotional regulation [90]. The cumulative effect of these neuroplastic changes is particularly relevant in mitigating the cognitive decline associated with recurring depressive episodes, which are often correlated with a higher risk of developing dementia later in life [108,109]. Therefore, by promoting neurogenesis and enhancing neuroplasticity, antidepressants offer a dual benefit: alleviating the immediate symptoms of depression while simultaneously providing a long-term protective buffer against the cognitive decline and neuronal degeneration associated with both aging and neurodegenerative diseases [90,108,109,110].

We would also like to mention one large-scale retrospective analysis, which included over 1.4 million individuals, that found that those who underwent long-term antidepressant treatment had a considerably lower chance of developing AD compared to those with shorter or no treatment [20,108,109]. In addition, randomized controlled trials have demonstrated that SSRIs like fluoxetine and sertraline can enhance cognitive functioning in patients with MCI and AD [90,110,111,112]. Fluoxetine, for instance, has shown significant positive effects on cognition in MCI patients, while sertraline has led to similar improvements in those with AD, highlighting the potential of SSRIs to counteract cognitive decline. Moreover, evidence from a longitudinal cohort study indicates that depressed MCI individuals who received long-term SSRI therapy (over four years) experienced a marked delay in the progression from MCI to Alzheimer’s disease [20,90,108,109,110,111,112,113].

Overall, the literature data supports the idea that long-term antidepressant treatment has dual benefits: it effectively alleviates depressive symptoms while also fostering neurogenesis and synaptic remodeling in crucial brain regions such as the hippocampus. By doing so, antidepressants minimize the chances of developing dementia among healthy populations and slow the rate of cognitive decline in those already experiencing early signs of dementia [90].

### 5.2. The Role of Antidepressants in Amyloid Accumulation

The investigation into the impact of antidepressants, particularly SSRIs, on amyloid burden in AD has provided compelling evidence, especially regarding their potential to mitigate amyloid plaque formation and improve cognitive outcomes [90]. Animal studies suggest that SSRIs may alter the APP processing pathway, steering it from a pro-amyloidogenic to a non-amyloidogenic route [114]. One significant example is citalopram, which has been shown to not only stop the progression of preexisting amyloid plaques but also reduce plaque load in mouse models. This decline in amyloid plaques correlates with a significant reduction in insoluble Aβ40 in the hippocampal and cortical regions, suggesting that SSRIs target key processes involved in amyloid pathology [114,115,116]. Furthermore, these findings are reinforced by human studies, including a prospective trial where healthy volunteers receiving acute doses of citalopram exhibited a notable reduction in amyloid-beta production in CSF. Specifically, the treatment group showed a 40% decrease in total CSF Aβ levels compared to placebo. This inhibition of Aβ oligomer aggregation points to an essential mechanism responsible for the neuroprotective effects of SSRIs, offering promising therapeutic strategies to combat AD pathology [90,114,115,116,117,118].

Other classes of antidepressants have also demonstrated promising neuroprotective effects in the context of AD, particularly in relation to amyloid accumulation and toxicity [90]. Tranylcypromine (MAOIs) has been shown to shield cortical neurons from the toxic effects of synthetic Aβ1–42 oligomers, preventing neuronal death and modulating the early stages of Aβ aggregation [114]. Also, fluoxetine has been demonstrated to prevent the increase in amyloid-beta levels in transgenic mouse models of AD, offering a protective effect over time against amyloid accumulation. Amitriptyline, a tricyclic antidepressant, further underscores the neuroprotective potential of antidepressants through its ability to inhibit the Aβ1–42-induced activation of extracellular signal-regulated kinase 1 and 2 (ERK1/2). By blocking the activation of these kinases, amitriptyline mitigates Aβ-induced neurotoxicity [114,115,116]. Additionally, research into the pharmacodynamics of antidepressants has revealed that pretreatment with amitriptyline can modify the epigenetic landscape of neurons [90,117]. This alteration induces gene expression changes that help counteract neuronal cell death, adding another layer of neuroprotection and highlighting the potential of antidepressants in modulating not only the biochemical but also the genetic pathways involved in AD pathology [90,114,115,116,117,118].

### 5.3. The Role of Antidepressants in Tau Pathology

Beyond their impact on amyloid pathology, antidepressants may also influence tau pathology, another key feature of AD represented by the accumulation of hyperphosphorylated tau protein in neurofibrillary tangles [90]. Emerging animal research has provided evidence that SSRIs, particularly escitalopram, may have the potential to reduce tau hyperphosphorylation, which could help alleviate tau-related neurodegenerative processes [119]. Studies conducted in rodent models of AD have demonstrated that escitalopram treatment can ameliorate both tau hyperphosphorylation and the associated spatial memory impairment. This effect appears facilitated by the activation of the Akt/GSK-3β signaling pathway, a critical intracellular pathway that governs tau phosphorylation levels. Specifically, Akt phosphorylation inhibits glycogen synthase kinase-3β (GSK-3β), an enzyme that is central to the pathological hyperphosphorylation of tau. By restoring balance in this signaling cascade, antidepressants like escitalopram may help mitigate tau pathology and its detrimental cognitive consequences [90,119].

Despite these promising findings, research on the effect of antidepressants on tau pathology is still in its early stages. Most of the available evidence comes from preclinical animal studies, and there is a significant gap in understanding how these mechanisms apply to human populations. Moreover, the complexity of tau pathology, with its numerous phosphorylation sites and interacting molecular pathways, highlights the need for more detailed investigations [90,119,120].

### 5.4. The Role of Antidepressants in Inflammation

The anti-inflammatory properties of antidepressants have gained significant attention in the context of the well-documented association between inflammation, depression, and dementia, that we described in detailed above [90]. Animal studies have shown that prolonged treatment with various antidepressants, such as fluoxetine, imipramine, and tianeptine, can effectively reverse stress-induced elevations in pro-inflammatory cytokines. These antidepressants seem to target inflammatory processes involving microglia and astrocytes, thereby promoting the downregulation of genes that contribute to pro-inflammatory responses [121,122,123]. For example, fluoxetine has demonstrated efficacy in reducing the expression of pro-inflammatory markers, including interleukin-6 (IL-6) and the nuclear factor kappa-light-chain-enhancer of activated B cells’ (NF-κB) signaling pathways, in rat models [90]. Considering the connection between inflammation and both depression and dementia, along with the high risk of progression from MCI to dementia in individuals with elevated pro-inflammatory cytokines, the early initiation of antidepressant treatment could be a vital approach for dementia prevention in specific cases [90,121,122,123].

## 6. Cognitive Risks of Antidepressant Use in Dementia

While antidepressants are undeniably effective in mitigating the emotional and psychological burden of depression, their long-term impact on dementia risk introduces a layer of complexity to treatment strategies. If depression contributes causally to the onset of dementia, it stands to reason that addressing depressive symptoms through pharmacological intervention should, in theory, reduce this risk [90]. However, the diverse pharmacodynamics of antidepressants, each influencing a distinct set of neurobiological pathways, complicates this assumption. These medications interact with various physiological systems implicated in both depression and dementia, mechanisms that we discussed earlier, such as neuroinflammation, neurovascular function, and the regulation of neurotrophic factors [104,105,106,107]. Consequently, while certain antidepressants may offer neuroprotective benefits by modulating these pathways, others might inadvertently exacerbate dementia risk through mechanisms unrelated to their efficacy in treating depression. This duality underscores the need for a nuanced, individualized approach to antidepressant therapy in older adults, where both the mental health benefits and the potential cognitive risks must be carefully weighed [90,104,105,106,107].

One notable example of how antidepressants can influence neurocognitive decline is through the anticholinergic side effects associated with certain classes of antidepressants, particularly tricyclic antidepressants (TCAs). Medications like amitriptyline, clomipramine, and doxepin have been shown to significantly elevate the risk of dementia, with research indicating that patients prescribed anticholinergic drugs are 1.26 times more likely to develop dementia compared to those who do not use such medications. This association is particularly concerning due to the well-documented severe side effects, including postural hypotension and urinary retention, which present further safety risks in older adults. Furthermore, it is worth mentioning that TCAs block muscarinic acetylcholine receptors in the CNS, key players in cognitive function, further amplifying the risk of neurocognitive decline [12,28]. Considering these risks, the 2023 American Geriatrics Society Beers Criteria strongly advises against the use of antidepressants with potent anticholinergic properties, such as amitriptyline, amoxapine, clomipramine, desipramine, doxepin, imipramine, nortriptyline, and paroxetine, in older adults with dementia or cognitive impairment. By contrast, other antidepressant classes, including SSRIs like fluoxetine, SNRIs such as venlafaxine, atypical antidepressants (such as bupropion, trazodone, and mirtazapine), and MAOIs like moclobemide, are generally considered safer for older adults. These alternatives present fewer anticholinergic effects, thereby reducing the risk of exacerbating cognitive decline [12,28,124,125,126].

Despite the conflicting findings in the existing literature, a comprehensive observational case–control study was conducted to clarify the correlation between antidepressant use and the likelihood of developing dementia, and its findings are particularly eloquent and noteworthy. This study offers meaningful observations of the complex interactions between antidepressant medications and cognitive decline, making it a significant contribution to understanding the nuanced risks and advantages of these treatments in this vulnerable population. The study analyzed data from over 62,000 older adults diagnosed with dementia, each matched with a control of similar size, creating a robust dataset. What set this research apart was its detailed classification of antidepressants, while also considering the duration of treatment. The results provided nuanced insights, revealing that the risk of dementia varied depending on the class of antidepressant. Notably, the use of SSRIs and SNRIs was associated with a heightened likelihood of dementia, while TCAs and several herbal remedies appeared to reduce this risk. Among SSRIs, citalopram stood out as a drug statistically linked to an increased risk of dementia, a finding also observed with mirtazapine [22,72,90,127,128,129,130].

The analysis further revealed a clear distinction between short- and long-term antidepressant use, highlighting a complex relationship between duration and dementia risk. Notably, long-term antidepressant use was associated with a reduced risk of dementia compared to short-term use, with escitalopram presenting a particular counterexample. In this case, short-term treatment with escitalopram was paradoxically linked to an increased risk of dementia, whereas long-term use decreased that risk. Interestingly, the reduced dementia risk associated with long-term use might also reflect early-onset depression, a form of the illness that may be less correlated with dementia development [22,28,72,130]. Despite these nuanced findings, the overall lower odds of dementia observed with the prolonged use of most antidepressants suggest that these medications do not inherently contribute to cognitive decline. However, the unexpected inverse relationships seen with certain drugs, such as escitalopram, also hint at the possibility of unadjusted confounding factors. However, the inherent limitations of observational studies complicate the interpretation of these findings, particularly when the reasons for varying treatment durations remain unclear. For instance, if a patient’s depressive symptoms naturally remit while they continue antidepressant therapy, it may misleadingly appear that the medication is responsible for the improvement, rather than the natural progression of their condition [22,72,131]. Additionally, if short-term antidepressant treatment is initiated in patients already exhibiting early signs of dementia, this could artificially inflate the perceived risk of dementia linked to these drugs. These complexities highlight the difficulty of separating the effects of antidepressants from the natural progression of depression and cognitive decline, underscoring the need for the careful interpretation of these results. Understanding the reasons behind treatment duration is essential for accurately assessing the true relationship between antidepressant use and dementia risk [22,28,72,130,131,132].

When considering biases, it is important to acknowledge that clinicians often select antidepressants based on perceived side effects, especially cognitive impacts. This can inadvertently affect the observed associations between antidepressants and dementia risk. For instance, providers may favor SSRIs over TCAs for patients at risk of cognitive decline, potentially leading to a misleading appearance of increased dementia risk with SSRIs and a reduced risk with TCAs [22,72]. Severe or treatment-resistant depression, which is independently correlated with an increased risk of dementia, may also affect treatment decisions. More complex cases tend to receive pharmacological interventions, while milder cases may be treated with non-pharmacological approaches. This variability in treatment approaches could obscure the true relationship between antidepressant use and dementia risk. In instances of severe depression, cognitive decline may occur alongside depressive symptoms, complicating accurate dementia diagnoses and potentially distorting study outcomes [22,72,128,129,130,131,132,133,134].

## 7. Treatment-Resistant Depression in Dementia

The phenomenon of treatment resistance to antidepressants in individuals with dementia presents a complex challenge, deeply intertwined with the neurodegenerative processes that underlie both cognitive decline and mood regulation [18,19]. Central to this issue is the disruption of key neurotransmitter systems, such as serotonin and norepinephrine, which are not only crucial for mood stabilization but also heavily influenced by the neurobiological alterations associated with dementia. As dementia progresses, the brain’s capacity for neuroplasticity, the ability to form and reorganize synaptic connections, diminishes, further reducing the efficacy of antidepressants, which often rely on enhancing neuroplastic mechanisms to alleviate depressive symptoms [18,19,135]. Additionally, dementia itself, with its progressive erosion of memory, executive function, and identity, creates a profound emotional burden, leading to a form of depression that may be more resistant to conventional antidepressant therapies. This intricate interplay of neurochemical, cognitive, and emotional factors highlights the need for a more tailored approach to managing depression in individuals with dementia, one that considers the unique neurophysiological landscape of the aging brain. We will briefly discuss several key mechanisms related to treatment resistance to antidepressants in individuals with dementia that we find particularly important to mention [18,19,135,136,137,138,139].

In neurocognitive disorders, particularly Alzheimer’s disease, the serotonergic system undergoes substantial degeneration, severely affecting mood regulation and cognitive functions. The dorsal raphe nucleus, the primary site for serotonin synthesis, experiences significant neuronal loss. As the disease advances, research suggests that up to 77% of serotonergic neurons can be lost, drastically reducing serotonin levels in the brain [135,140]. This neurochemical imbalance not only contributes to the emotional and cognitive symptoms of dementia but also diminishes the effectiveness of selective serotonin reuptake inhibitors. Also, a key player in the regulatory mechanisms that control neurotransmitter activity is the serotonin-1A (5-HT1A) autoreceptor, which governs serotonergic signaling through feedback inhibition. Under normal conditions, these autoreceptors modulate serotonin release, maintaining balance within the system. However, in dementia, the distribution and functionality of 5-HT1A receptors become dysregulated [135,141,142]. For example, patients with vascular dementia have been demonstrated to exhibit increased 5-HT1A receptor binding, which can disturb serotonergic transmission and exacerbate symptoms such as agitation, anxiety, and depression. On the other hand, in Alzheimer’s disease, decreased 5-HT1A receptor binding is observed, impairing the brain’s capacity to regulate serotonin levels effectively [135]. Understandably, these alterations can reduce the responsiveness to SSRIs, which rely on functional serotonergic pathways to alleviate depressive symptoms [135,140,141,142,143,144].

The noradrenergic system, particularly centered around the locus coeruleus (LC), is another critical pathway significantly affected by dementia. The LC plays a key role in producing and distributing noradrenaline throughout the brain, which influences attention, emotional regulation, and memory. Research indicates that individuals with mild cognitive impairment can experience up to 30% neuronal loss in the LC, with this loss increasing to 55% in advanced dementia syndromes. This progressive decline in the LC correlates with worsening behavioral and psychological symptoms, including heightened anxiety, depression, and cognitive deficits. As the noradrenergic system deteriorates, antidepressants that target noradrenaline, SNRIs, may not deliver the expected therapeutic benefits [135,139,145,146].

Moreover, neuroplasticity is fundamental to the therapeutic effects of antidepressants. Many of these medications, particularly SSRIs, have been shown to enhance the expression of brain-derived neurotrophic factor (BDNF), a critical protein that supports neuronal health, survival, and synaptic plasticity. As we mentioned before, BDNF plays a pivotal role in enabling the brain to adapt to stress and recover from depressive symptoms by fostering neuroplastic changes in key regions like the hippocampus. However, in the context of dementia, BDNF levels are often significantly reduced due to ongoing neurodegeneration. This decline severely limits the brain’s ability to undergo the neuroplastic adaptations necessary for recovery from depression, thus reducing the effectiveness of traditional antidepressants [135,147,148,149,150].

Given the intricate interplay of neurobiological factors contributing to treatment resistance in dementia-related depression, a more personalized approach to treatment becomes essential. Clinicians must evaluate not only the neurochemical imbalances but also consider the degree of cognitive decline and the patient’s neuroplastic potential when selecting antidepressant therapies. Traditional medications may have diminished efficacy due to neurodegeneration in key regions, and thus, alternative treatment strategies are critical. Psychotherapy, though challenging in dementia due to cognitive impairments, might still offer benefits, especially when adapted for patients with mild cognitive impairment. Furthermore, novel pharmacological interventions aimed at enhancing neuroplasticity, such as agents that increase BDNF levels, hold promise for improving both mood and cognitive function in this population [135,136].

## 8. Discussion

The relationship between depression and dementia is highly complex, particularly in older adults. As mentioned earlier, untreated or recurrent depressive episodes are significant risk factors for the development and progression of neurocognitive disorders. This is driven by various interconnected factors, such as vascular changes, neuroinflammation, and disruptions in neurotrophic pathways, which not only accelerate cognitive decline but also exacerbate depressive symptoms. Therefore, it is crucial to address depression in older individuals, not only to alleviate mood symptoms but also to preserve cognitive function [12,19,150].

In this context, antidepressants have demonstrated a range of neuroprotective effects that go beyond symptom relief, potentially slowing the trajectory of cognitive decline. A key component of this neuroprotection is the ability to promote neurogenesis and enhance synaptic plasticity, particularly within the hippocampus. Antidepressants like fluoxetine and venlafaxine stimulate the proliferation of neural progenitor cells and strengthen synaptic connections, thus counteracting the neural atrophy associated with both depression and early-stage dementia. This neurogenic effect is driven by the upregulation of BDNF signaling, which supports neuronal survival and synaptic resilience, offering a potential buffer against the cognitive deterioration typically linked to neurodegenerative diseases [30,31,32,151,152,153].

Antidepressants, beyond their neurogenic effects, have emerged as important modulators of neuroinflammation, which is a critical factor in both depression and dementia. Chronic neuroinflammation, marked by elevated pro-inflammatory cytokines such as IL-6 and TNF-α, plays a pivotal role in accelerating cognitive decline. Antidepressants like fluoxetine and sertraline have been shown to downregulate these inflammatory pathways, reducing the production of harmful cytokines while promoting anti-inflammatory mediators. This creates a more favorable environment for neuronal repair, potentially slowing the progression of both depressive symptoms and cognitive deterioration in dementia patients [30,31,32,58,72].

Emerging evidence further suggests that antidepressants may directly modulate key pathological features of AD, such as Aβ build-up and tau hyperphosphorylation. SSRIs like citalopram have been shown to influence the APP processing pathway, steering it away from amyloidogenic routes, which reduces the production and accumulation of Aβ plaques. This shift is particularly significant given the central role of amyloid plaques in AD pathology, positioning certain antidepressants as potential disease-modifying agents [73,90,152,153,154].

However, despite these promising mechanisms, a significant challenge in this therapeutic landscape is the issue of treatment resistance, which complicates the management of both depression and cognitive decline in dementia patients. In such cases, the neuroprotective and mood-alleviating effects of standard antidepressant therapies may be insufficient. Treatment-resistant depression in dementia patients may be driven by several factors, including advanced neurodegenerative changes that render the brain less responsive to pharmacological interventions, chronic inflammation, and altered neurotransmitter dynamics. This highlights the need for novel therapeutic approaches that go beyond traditional antidepressant regimens, possibly incorporating strategies that target both mood and cognitive symptoms through alternative mechanisms [12,19,30,31,32,151,152,153,154,155].

Although some evidence suggests that long-term antidepressant use may reduce the odds of developing Alzheimer’s disease, other studies indicate a potential increase in risk, particularly with short-term or early-stage use in patients who may already be experiencing undiagnosed cognitive decline. This discrepancy could be explained by the heterogeneity in patient populations, treatment durations, and the underlying depressive pathology. For example, severe depression, which is independently associated with a higher risk of dementia, may lead to increased antidepressant use, thereby confounding the observed associations between medication and cognitive outcomes. Additionally, the initiation of antidepressants in individuals with prodromal dementia may create a misleading impression that these medications contribute to cognitive decline when, in fact, they are being prescribed in response to early cognitive symptoms [19,30,72,90].

We believe that the conflicting evidence in studies examining the relationship between antidepressant use and dementia risk primarily stems from the heterogeneity of their designs, especially within observational studies, which dominate the literature on this topic. One of the key challenges in interpreting the findings from these studies is the presence of numerous confounding variables, which are often either unaccounted for or inadequately adjusted in the analysis. These confounders can independently contribute to cognitive decline, making it extremely difficult to isolate the specific effects of antidepressants on cognitive function. As a result, the heterogeneity across studies is often high, leading to inconsistent and at times contradictory findings [12].

Furthermore, many of these studies are limited by several factors that prevent them from providing definitive answers. For example, cumulative exposure to antidepressants is often not evaluated in sufficient detail, leaving a major gap in understanding how the long-term use of these medications may influence cognitive outcomes [104,105]. Additionally, the progression of depression itself could explain some of the observed long-term risks of dementia in antidepressant users, but much of the research does not fully capture this critical aspect. Many studies also fail to distinguish between different types of dementia beyond Alzheimer’s disease, such as frontotemporal dementia or Lewy body dementia, despite the possibility that antidepressants may have varying effects across these different forms of neurodegeneration. This lack of specificity further complicates the interpretation of the available data [12,104,105].

The inconsistency across studies also explains the absence of definitive clinical guidelines or recommendations for managing antidepressant therapy in individuals with dementia or those at risk of developing dementia, leaving clinicians without a reliable framework to guide their treatment decisions. The lack of robust, evidence-based guidelines is particularly concerning given the potential risks associated with antidepressant use in older adults [12].

We consider that future research must aim to fill these gaps by more effectively controlling for confounding factors, especially depression severity and comorbidities. It is crucial that standardized clinical scales are used in order to assess depression severity, and that cumulative exposure to antidepressants over time is better accounted for. Future studies should also consider the potential differential effects of antidepressants on various types of dementia and explore the specific impact of antidepressant use on cognitive outcomes [105,106]. Moreover, researchers need to investigate whether antidepressants may influence cognitive decline differently depending on the stage of dementia or the severity of depression at baseline. Until these issues are resolved, the evidence surrounding antidepressants and dementia risk will remain inconclusive, and healthcare providers will continue to lack clear guidelines on how to navigate this complex therapeutic dilemma. In the absence of well-defined recommendations, clinicians will be forced to make individualized decisions, weighing both the potential mental health benefits and cognitive risks of antidepressant therapy in older adults [12,104,105,106,107].

## 9. Conclusions

In conclusion, the role of antidepressants in dementia is multifaceted, extending well beyond their primary purpose of treating depression. They offer potential neuroprotective effects through neurogenesis, anti-inflammatory activity, and the modulation of amyloid and tau pathologies, suggesting a promising approach to cognitive preservation [12,22,28]. However, the risk of developing dementia after antidepressant use remains a concern, with some studies indicating an increased risk, especially in older adults. This ambiguity in the data underscores the need for more well-designed randomized controlled trials to clarify long-term cognitive outcomes and better understand treatment resistance. Supporting new studies is crucial, because the current literature is inconclusive, and a more personalized approach to therapeutic strategies is necessary to address the unique challenges faced by this population. We find future research essential in developing more effective, targeted interventions that not only alleviate depressive symptoms but also slow the progression of cognitive decline, offering hope for a comprehensive treatment strategy in managing these interrelated disorders [12,22,28,58,72].

## Figures and Tables

**Table 1:** Research findings on the connection between dementia and depression [31].

	Supporting Evidence	Opposing Evidence
Can depression contribute to the development of dementia?	Systematic reviews and meta-analyses indicate a higher risk of AD in individuals with a history of depression [74]. Several longitudinal studies have linked LLD with an increased risk for all-cause dementia [75]. Long-term cohort studies also support that depression, particularly recurrent episodes, is correlated with a greater likelihood of developing dementia [76].	Some community-based cohort studies find no significant association between mood disturbances and the onset of dementia [77]. Certain population-based studies over long periods did not find depression as a strong predictor of dementia development [78].
Is depression a prodromal state of dementia?	Longitudinal cohort studies indicate that late-life depression, especially when it begins early, might serve as an early indicator or prodromal stage of dementia [79]. Additionally, research shows that late-life depression could be part of the early stages of Alzheimer’s disease [80].	Some community-based studies have found no significant evidence that mood disturbances in early life are linked to a heightened risk of developing dementia [81].
Can depression aggravate the neurocognitive deficit?	Cohort studies have indicated that individuals with dementia who also experience depressive symptoms tend to experience more rapid cognitive decline. This suggests that depression may play a role in accelerating neurodegeneration [82].	Some studies have found no indication that the severity of cognitive decline is affected by the onset of depression [67].
Is depression a consequence of cognitive decline?	Longitudinal studies have found that as memory performance worsens, it can predict future increases in depressive symptoms. This suggests that depression may be a psychological response to cognitive decline, rather than a risk factor for dementia [67].	Some studies have found no evidence that cognitive decline affects the onset of depression, regardless of whether the individual has a dementia diagnosis or mild cognitive impairment (MCI) [68].

**Table 2:** Various antidepressants and their neuroprotective effects [90].

Antidepressant	Neuroprotective Mechanism
Fluoxetine	Gene Regulation in Inflammation (Fluoxetine downregulates several genes involved in pro-inflammatory pathways, including IL-6, NF-κB, and TNF, which are key players in chronic inflammatory states.) Prevention of Amyloid-β Accumulation (This SSRI reduces the accumulation of amyloid-β, a protein associated with Alzheimer’s disease pathology.) Neurogenesis and Synaptic Plasticity (Fluoxetine promotes the remodeling of synapses and enhances neurogenesis in the hippocampus, partly by activating the CREB-BDNF signaling pathway. It also reverses the negative impact of chronic stress on hippocampal neurogenesis.)
Citalopram	Amyloid-β Suppression (Citalopram suppresses the generation of amyloid-β peptides, particularly in the hippocampus and cortical regions. This results in a decrease in the levels of insoluble Aβ40, which is a key factor in plaque formation in Alzheimer’s disease.)
Escitalopram	Tau Protein Modulation:(Escitalopram helps to prevent the hyperphosphorylation of tau proteins, which is triggered by factors like Aβ1–42. It does this through the activation of the PKA pathway and the stimulation of the 5-HT1A receptor, which in turn regulates Akt/GSK-3β signaling, key in tau pathology and Alzheimer’s.)
Venlafaxine	Anti-inflammatory Action (This SNRI promotes the release of TGF-β, an anti-inflammatory cytokine, and simultaneously reduces the secretion of pro-inflammatory markers like IL-6 and IFN-γ.) Microglial Phenotype Shift (Venlafaxine changes microglia from an activated, inflammatory state to a more resting, non-inflammatory morphology.)
Bupropion	Inflammatory Cytokine Reduction (Bupropion, an NDRI, specifically reduces the production of pro-inflammatory cytokines like TNF-α and IFN-γ, both of which are involved in neuroinflammatory processes that can lead to neurodegeneration.)
Moclobemide	Cytokine Balance Regulation (Moclobemide, a reversible inhibitor of monoamine oxidase A (RIMA), downregulates pro-inflammatory factors like IL-1β and TNF-α while promoting anti-inflammatory cytokines like IL-10.) Neurogenesis Restoration (This drug reverses stress-induced reductions in neurogenesis within the hippocampus and protects neurons by inhibiting apoptotic pathways.)
Tranylcypromine	Prevention of Amyloid-β Toxicity (Tranylcypromine prevents the neurotoxic effects of amyloid-β (Aβ), particularly by reducing Aβ-induced neuronal cell death. It also inhibits the aggregation of Aβ proteins, which is crucial in preventing amyloid plaque formation in neurodegenerative diseases.)
Amitriptyline	Neuroprotection Against Amyloid Toxicity (Amitriptyline, a tricyclic antidepressant, exerts protective effects against Aβ1–42-induced neurotoxicity. It inhibits the activation of ERK1/2, a pathway associated with the harmful effects of Aβ on neurons, thus protecting them from amyloid-induced damage.)
